# Insufficient access to harm reduction measures in prisons in 5 countries (PRIDE Europe): a shared European public health concern

**DOI:** 10.1186/s12889-015-2421-y

**Published:** 2015-10-27

**Authors:** Laurent Michel, Caroline Lions, Sara Van Malderen, Julie Schiltz, Wouter Vanderplasschen, Karina Holm, Torsten Kolind, Felice Nava, Nadja Weltzien, Andrea Moser, Marie Jauffret-Roustide, Olivier Maguet, Patrizia M Carrieri, Cinzia Brentari, Heino Stöver

**Affiliations:** Inserm U1178, Paris, France; Univ Paris-Sud and Univ Paris Descartes, UMRS1178, Paris, France; Centre Pierre Nicole, French Red Cross, Paris, France; Inserm U912 (SESSTIM), Marseille, France; Univ Aix Marseille, IRD, UMR-S912, Marseille, France; ORS PACA, Observatoire Régional de la Santé Provence Alpes Côte d’Azur, Marseille, France; Penitentiary administration, Brussels, Belgium; Department of Special Education, Ghent University, Ghent, Belgium; Centre for Alcohol and Drug Research, Aarhus University, Aarhus, Denmark; Federserd, Padova, Italy; Penitentiary administration, Vienna, Austria; Inserm U988, Paris, France; Médecins du Monde, Paris, France; University of Applied Sciences, Frankfurt, Germany

**Keywords:** Prison, Prevention, HIV, HCV, Infectious diseases, Public health, Recommendations, Policy

## Abstract

**Background:**

Prisoners constitute a high-risk population, particularly for infectious diseases. The aim of this study was to estimate the level of infectious risk in the prisons of five different European countries by measuring to what extent the prison system adheres to WHO/UNODC recommendations.

**Methods:**

Following the methodology used in a previous French survey, a postal/electronic questionnaire was sent to all prisons in Austria, Belgium, Denmark and Italy to collect data on the availability of several recommended HIV-HCV prevention interventions and HBV vaccination for prisoners. A score was built to compare adherence to WHO/UNODC recommendations (considered a proxy of environmental infectious risk) in those 4 countries. It ranged from 0 (no adherence) to 12 (full adherence). A second score (0 to 9) was built to include data from a previous French survey, thereby creating a 5-country comparison.

**Results:**

A majority of prisons answered in Austria (100 %), France (66 %) and Denmark (58 %), half in Belgium (50 %) and few in Italy (17 %), representing 100, 74, 89, 47 and 23 % coverage of the prison populations, respectively. Availability of prevention measures was low, with median adherence scores ranging from 3.5 to 4.5 at the national level. These results were confirmed when using the second score which included France in the inter-country comparison. Overall, the adherence score was inversely associated with prison overpopulation rates (p = 0.08).

**Conclusions:**

Using a score of adherence to WHO/UNODC recommendations, the estimated environmental infectious risk remains extremely high in the prisons of the 5 European countries assessed. Public health strategies should be adjusted to comply with the principle of equivalence of care and prevention with the general community.

## Background

“Prison health is public health” [1]. The burden of illnesses, particularly infectious diseases, is heavier in the prison population than in the general population, with high prevalences of HIV, HCV and tuberculosis, as well as higher rates of mental disorders, including drug/alcohol use disorders [2]. The risk of prisoners transmitting disease is high as they are in constant contact with the general community through visitors, staff and because a considerable proportion of them can cycle in and out of prison. National strategies to control transmission risks should include prisons [3]. Despite being adopted in several countries, the principle of equivalence in health, including prevention, between the prison system and the general community is rarely implemented in reality [4, 5]. For example, although France adopted the principle in 1994, the ANRS-PRI^2^DE survey (2009–2010), which measured the level of adherence to national and WHO/UNODC guidelines, revealed a gap between the official recommendations and their application in French prisons [6]. Data on health policy implementation in general in the prison setting are sparse, and conducting research to explore related issues is difficult [7–9].

The main objective of this international survey was to compare the levels of environmental infectious risk in prisoners between 4 different countries, by measuring to what extent prisons in these countries adhere to international recommendations for HIV prevention. This measurement was achieved by computing an adherence score which was modified to take into account the latest international recommendations. We also aimed to create a 5-country comparison, in order to include France, by creating a second adherence score.

The study’s secondary objective was to identify which particular characteristics of prison settings and penitentiary policies are correlated with non-adherence to WHO/UNODC recommendations.

## Methods

### Data collection

A nationwide survey targeting all prisons in each of the following 4 European countries was conducted between June 2013 and April 2014: Belgium (35 prisons), Austria (28 prisons), Denmark (50 prisons) and Italy (205 prisons). A previous survey in France was conducted between November 2009 and May 2010 and targeted all French prisons (170 prisons).

In order to estimate "environmental" infectious risk, an electronic/postal survey questionnaire focusing primarily on the availability of HIV-HCV preventive measures and HBV vaccination for prisoners was sent to the heads of medical units in all the prisons in all 5 countries (in 2009–2010 for France, in 2013–2014 for the other 4 countries).

More specifically, the questionnaire collected data about the structural characteristics of the prisons and assessed the availability of different specific prevention measures. The dimensions explored were related to the 10 following interventions recommended by WHO/UNODC [10, 11]: Bleach, Condoms and lubricants, Opioid Substitution Treatment (OST), Information-Education-Communication (IEC), Blood-borne virus (BBV) testing, HBV vaccination, Post-exposition Prophylaxis (PEP), Needle/syringe exchange programs (NEP), access to ARV and prevention measures for tattooing/piercing. Data on prison characteristics included: 1) type of structure (remand centre, prison, security prison and juvenile prison – some prisons included several types of structures); 2) gender of the prison population (male and/or female inmates); 3) number of prisoners on the day of the study; 4) total numbers of sanitary staff, doctors, nurses, security staff, and social workers working in the prison; 5) number of full-time doctors and nurses working in the prison; 6) existence of consultation service for HIV, HCV/HBV and psychiatric consultation, and frequency of consultation (every week, every 2 weeks, etc.); 7) presence of any non-governmental organization (NGO) involved in harm reduction (HR) and care for drug users in the prison. We also collected the following data from the council of Europe’s annual penal statistics (Space project - http://wp.unil.ch/space/) which reflect penal policy at the national level: average national levels for penitential density for 100 places (this provides a measure of prison overpopulation), percentage of prisoners who were sentenced primarily for drug offences, prisoner security staff member ratio, and the ratio between prisoners and other professionals working in prison.

The survey was implemented in all 24-hour incarceration prisons at the national level (all semi-liberty prisons and all other penal alternatives to imprisonment were excluded). Juvenile detention centres receiving only minors were excluded.

### Definition of the scores of adherence:

We used two documents providing official international recommendations for interventions to prevent HIV and other infectious diseases’ transmission in prison settings to create adherence scores:

- The first was “Effectiveness of interventions to address HIV in prisons”, written by the WHO in 2007, in collaboration with UNAIDS and UNODC, which is the most detailed document on HIV prevention in prison settings [10]. The purpose of this recommendation was to document and define the conditions required to implement all interventions with proven efficacy for HIV- and HCV-transmission prevention in prison settings.
- The second document was “HIV prevention, treatment and care in prisons and other closed settings: a comprehensive package of interventions”, written by the UNODC in 2012, in collaboration with UNAIDS, WHO, ILO and UNDP. It describes 15 key interventions to prevent HIV and viral hepatitis transmission in prison settings [11]. It is shorter than its WHO counterpart, but lists all current (as of 2015) interventions to implement in prison settings, except bleach, whose efficacy in preventing HIV-HCV in the prison context has not been proven. In particular, it describes three important measures, among others, which were not clearly detailed in the 2007 WHO document: access to ARV, HBV vaccination and prevention measures for tattooing and piercing.

In order to evaluate the implementation of HIV prevention and other HR measures in prisons and to estimate the level of environmental infectious risk exposure in the 5 different countries investigated, two scores of adherence to HIV preventive measures were built: the first, using the second document above, reflected the most recent recommendations for adherence to WHO/UNODC recommendations (2012), and ranged from 0 (no adherence) to 12 (full adherence). It was used to compare adherence between Austria, Denmark, Belgium and Italy. The second score, constructed to create a 5-country comparison, included data from the 2009–2010 French ANRS-PRI^2^DE survey and ranged from 0 to 9. This different scoring system was required because at the time of the French survey, only the 2007 WHO recommendations (first document above) were available and did not include the following 3 recommendations: accesss to ARV, HBV vaccination and prevention measures for tattooing and piercing.

Table 1 shows items corresponding to international recommendations and how adherence to each specific recommendation was scored. For each of the two adherence scores constructed, a global score was built, by summing the subscores corresponding to each recommendation. Each subscore ranged from 0 to 1 (i.e., 0, 0.5, 1) with the exception of the scores for opioid substitution treatment and access to condoms. Subscores for these two measures ranged between 0 and 2, in order to give them a higher weighting, given that their effectiveness in HIV prevention is considered very high [10]. Therefore, potential overall scores for adherence ranged from 0 to 12 (10 recommendations) for the 2013 survey for Austria, Belgium, Denmark and Italy, and from 0 to 9 (7 recommendations) for the 2009 French survey (as the 3 abovementioned recommendations were not assessed). For each subscore, we computed the proportion of prisons in each country adherent to international recommendations. In line with the 2007 WHO recommendations, we set the subscores for IEC and Testing/counseling at zero in both surveys, as NEP was not available in all prisons (“*prisoners must be provided with the prevention measures that enable them to act upon the information they receive, such as condoms and clean injecting equipment*”). In the first analysis, which compared adherence in Austria, Belgium, Denmark and Italy, only global scores (ranging from 0 to 12) were taken into account (hereafter Europe 4 analysis or E4). In the second analysis, which included data from the 2009 French survey to create a 5-country comparison , global scores (ranging from 0 to 9) were used, (hereafter Europe 5 analysis: E5).

**Table 1:** Scoring method for computing adherence to international recommendations in prisons (PRIDE Europe)

	International Recommendations	Score
Information-Education-Communication	•Availability of Information/education at entry or during prison stay	0.5
	•Peer education programs available	0.5
	•AND availability of clean injecting equipment + condoms (0 if not)^a^	**1**
Testing - Counseling	•Testing for HIV, HBV, HCV systematically proposed at entry (RC) and during prison stay (all prisons)	
	•AND availability of clean injecting equipment + condoms (0 if not)*	**1**
Condoms - Lubricants	•Condoms available in various locations	1
	•Water-based lubricants available	0.5
	•Male condoms and lubricants accessible and female condoms accessible for prisons with female prisoners	0.5
		**2**
Opioid Substitution Therapy	•Induction at entry (RC) + induction during prison stay + continuity of OST at entry (all prisons)	1
	•No ceiling dosage	0.5
	•No buprenorphine crushing or dilution	0.5
		**2**
Bleach	•At least 2 locations/access for bleach inside prison (penitentiary distribution, purchasable inside prison, available in medical unit)	
	•AND Intelligible information for HR purposes accessible for all prisoners	**1**
HBV Vaccination^b^	•Systematic HBV vaccination proposal for all seronegative prisoners	**1**
Post-Exposition Prophylaxis	•All prisoners informed of PEP availability inside prison	**1**
Needle Exchange Programs	•NEP are available	**1**
ARV treatment^b^	•ARV are accessible	0.5
	•Prescriptions follow national guidelines	0.5
		**1**
Prevention of transmission through tattooing, piercing^b^	•Existing initiatives aiming at reducing the sharing and reuse of equipment used for tattooing, piercing and other forms of skin penetration	**1**
TOTAL		**12**

^a^Condition defined in the 2007 WHO recommendations for IEC and Testing/counseling: “prisoners must be provided with the prevention measures that enable them to act upon the information they receive, such as condoms and clean injecting equipment”. ^b^These interventions were not included in the international scoring calculation in the 2009 French ANRS-PRI^2^DE survey

Bold numbers are the total value for each subscore

### Statistical analysis

In the E4 analysis, a Chi-square test or Fisher exact test (when at least one cell contained fewer than 5 observations), was used to measure the association between adherence to each of the 10 interventions recommended by WHO/UNODC. The score as a continuous variable was also compared using the non-parametric Kruskal-Wallis test.

Univariate linear regression models were used to assess the impact of each prison characteristic and national penal characteristics (e.g. the average number of prisoners per security staff member) on the level of adherence to international recommendations.

As there is no participation of human subjects in the “PRIDE Europe” project, the Good Clinical Practice (international ethical and scientific quality standard for designing, conducting, recording and reporting trials that involve the participation of human subjects – ICH Guideline) are not applicable and ethic committee approvals were unnecessary.

## Results

In the 2013–2014 survey, the participation rate (number of prisons) in each of the 4 countries was as follows: 50 % (17/34) in Belgium, 100 % (28/28) in Austria, 58 % (29/ 50) in Denmark and 17 % (35/205) in Italy, representing, respectively, 47, 100, 89 and 23 % of the total prison population (total prison population on 1st January 2014, according to the council of Europe’s annual penal statistics).

In the 2009 French survey, 113/171 prisons answered the questionnaire (66 %), representing 74 % of the prison population at that time.

Prisons with incomplete questionnaires or incoherent data were systematically called by phone, and those with missing data were not included in the data analysis.

The number of prisons with complete data for all the questionnaire items was as follows: 11, 19, 29, 35 and 103 for Belgium, Austria, Denmark, Italy and France, respectively. Table 2 shows the characteristics of the prisons at the national level for the E4 analysis only. Considering the internal validity of the study, the geographic distribution of prisons which responded was comparable to that of prisons which did not.

**Table 2:** E4 analysis- Characteristics of the prisons at the national level

	Belgium (N = 17)	Austria (N = 27)	Denmark (N = 29)	Italy (N = 35)
number of prisoners on the day of the study	6046	8724	3361	14229
number of prisoners on the day of the study per prison - Mean (min-max)	356 (50–1126)	312 (58–1146)	116 (14–489)	407 (30–1519)
number of prisoners on the day of the study per prison- n(%)				
<100	2 (11.8)	3 (10.7)	17 (58.6)	5 (14.3)
[100-350[	9 (52.9)	14 (50.0)	11 (37.9)	15 (42.9)
[350-600[	2 (11.8)	9 (32.1)	1 (3.4)	9 (25.7)
≥600	4 (23.5)	2 (7.1)	0 (0.0)	6 (17.1)
Number of personnel available / prison - Mean (min-max)				
Sanitary staff	14 (3–48)	10 (2–69)	5 (1–36)	24 (6–75)
Doctors	3 (1–7)	5 (1–28)	2 (1–7)	14 (3–43)
Nurses	10 (1–36)	5 (1–44)	3 (0–19)	10 (2–34)
Security staff	254 (42–707)	109 (22–421)	77 (11–460)	211 (30–950)
Social workers	7 (1–18)	4 (1–12)	2 (1–8)	5 (1–17)
Number of personnel available/100 prisoners - Mean (min-max)				
Sanitary staff	4 (2–12)	4 (1–43)	5 (2–18)	8 (2–37)
Doctors	1 (0.4-6)	2 (0.4-8)	2 (0.4-7)	5 (1–23)
Nurses	3 (1–6)	2 (0.2-28)	1 (0–5)	3 (1–13)
Security staff	70 (48–86)	37 (27–74)	64 (34–170)	29 (17–160)
Social worker	2 (0.4-7)	2 (1–7)	3 (0.4-9)	2 (1–3)
Available Consultation- n(%)				
HIV (yes vs. no)	2 (11.8)	28 (100)	28 (95.6)	30 (85.7)
HCV/HBV (yes vs. no)	2 (11.8)	28 (100)	28 (95.6)	30 (85.7)
Psychiatric (yes vs. no)	14 (82.4)	26 (92.9)	25 (86.2)	35 (100)
Attendance of any NGO (yes vs. no)	7 (50)	5 (19.2)	0 (0)	11 (31.4)
Type- n(%)				
RC only	5 (29)		16 (55)	30 (86)
PS only	5 (29)	10 (36)	3 (11)	5 (14)
SPS only			1 (3)	
JUV only				
RC-PS	5 (29)	4 (14)	3 (11)	
PS-JUV		2 (7)		
PS-SPS			1 (3)	
RC-SPS			4 (14)	
RC-PS- SPS	2 (12)		1 (3)	
RC-PS- SPS-JUV		12 (43)		
Gender- n(%)				
Male	12 (71)	13 (46)	3 (10)	18 (56)
Female	1 (6)	1 (4)	0	0
Mixed	4 (24)	14 (50)	26 (90)	14 (54)

HIV: Human Immunodeficiency Virus; HCV: Hepatitis C Virus; HBV: Hepatitis B Virus; NGO: Non-Governmental Organization; RC: remand center; PS: prison for sentenced; SPS: Security prison for sentenced; JUV: juvenile prison center

### Descriptive results

#### E4 analysis

Table 3 shows the proportion of prisons, in the E4 analysis, adhering to the international recommendations for each subscore making up the global adherence score.

**Table 3:** E4 analysis- proportion of adherence to the different subscores among all participating prisons (n = 109)

	Belgium (N = 17/35)	Austria (N = 28/28)	Denmark (N = 29/50)	Italy (N = 35/205)	*P* *
Information-Education-Communication	**0**	**0**	**0**	**0**	
• Availability of Information/education at entry or during prison stay	15 (88.2)	28 (100.0)	3 (10.3)	13 (37.1)	*<10* ^*−3*^
• Peer education programs available	4 (23.5)	2 (7.1)	4 (13.8)	1 (2.9)	*0.07*
• Availability of clean injecting equipment + condoms	0	0	0	0	
Testing – Counseling	**0**	**0**	**0**	**0**	
• Testing for HIV, HBV, HCV systematically proposed at entry (RC) and during prison stay (all prisons)	3 (17.7)	11 (39.3)	8 (27.6)	35 (100.0)	*<10* ^*−3*^
• Availability of clean injecting equipment + condoms	0	0	0	0	
Condoms - Lubricants	**9 (52.9)**	**8 (28.6)**	**2 (6.9)**	**0 (0.0)**	*0.002 ł*
• Condoms available in various locations	12 (70.6)	14 (50.0)	14 (48.3)	0 (0.0)	*0.29 ł*
• Water-based lubricants available	13 (76.5)	23 (82.1)	6 (20.7)	0 (0.0)	*<10-3 ł*
• Male condoms and lubricants accessible and female condoms accessible for prisons with female prisoners	14 (82.4)	14 (50.0)	13 (44.8)	0 (0.0)	*0.04 ł*
Opioid Substitution Therapy	**4 (23.5)**	**6 (21.4)**	**7 (24.1)**	**5 (14.3)**	*0.66*
• Induction at entry (RC) + induction during prison stay + continuity of OST at entry (all prisons)	11 (64.7)	20 (71.4)	24 (82.8)	12 (34.3)	*0.0001*
• No ceiling dosage	8 (47.1)	15 (53.6)	19 (65.5)	30 (85.7)	*0.05*
• No buprenorphine crushing or dilution	15 (88.2)	17 (60.7)	11 (37.9)	17 (48.6)	*0.002*
Bleach	**0 (0.0)**	**11 (39.3)**	**11 (37.9)**	**0 (0.0)**	*<10* ^*−3*^
• At least 2 locations/access for bleach inside prison (penitentiary distribution, purchasable inside prison, available in medical unit)	0 (0.0)	20 (71.4)	17 (58.6)	0 (0.0)	*<10* ^*−3*^
• Intelligible information for HR purposes accessible to all prisoners	4 (23.5)	13 (46.4)	12 (41.4)	0 (0.0)	*0.30 ł*
HBV Vaccination (systematic proposal for unprotected inmates)	**0 (0.0)**	**4 (14.3)**	**3 (10.3)**	**18 (51.4)**	*<10* ^*−3*^
Post-Exposition Prophylaxis (inmates informed of the availability of PEP)	**0 (0.0)**	**11 (39.3)**	**1 (3.5)**	**27 (77.1)**	*<10* ^*−3*^
Needle Exchange Programs	**0**	**0**	**0**	**0**	
ARV treatment	**14 (82.4)**	**27 (96.4)**	**29 (100.0)**	**33 (94.3)**	*0.11*
• ARV are accessible	17 (100.0)	27 (100.0)	29 (100.00)	33 (89.2)	*0.24*
• Prescriptions follow national guidelines	14 (82.4)	27 (96.4)	29 (100.0)	33 (94.3)	*0.11*
Prevention of transmission through tattooing, piercing	**2 (11.8)**	**0 (0.0)**	**5 (17.2)**	**0 (0.0)**	*0.01*
Total score, median (Q1-Q3)	4.0 (3.5-4.5)	4.5 (3–6.5)	4.0 (3.0-4.5)	3.5 (2.5-4)	0.0014

*Chi-square or exact Fisher test for categorical variables, Wilcoxon-Mann–Whitney test for continuous variables

*ł* Excluding Italy

HIV: Human Immunodeficiency Virus; HCV: Hepatitis C Virus; HBV: Hepatitis B Virus; NGO: Non-Governmental Organization; RC: remand center; PS: prison for sentenced; SPS: Security prison for sentenced; JUV: juvenile prison center; OST: opioid substitution therapy; HR: Harm Reduction; ARV: Antiretroviral Treatment

Bold numbers are only to identify the total score for each subscore and is not related to its statistical value and *P* values are on the right column

For IEC and Testing-Counseling interventions, adherence scores equaled zero for the 4 countries assessed, as no country respected the required condition of available clean injecting equipment. However, the availability of systematic HIV testing was different across countries, ranging from 18 % in Belgium to 100 % in Italy (p < 10-3). Female condoms were available in approximately half of the prisons detaining female prisoners in Austria and Denmark and in 82 % of similar prisons in Belgium (p = 0.04). Condoms were not available in any Italian prison. Bleach was not accessible in Italy. In Belgium, bleach or disinfecting tablets were provided in all prisons, but only at one location. The systematic proposal of HBV vaccination was rare, except in Italy, where it was proposed in half of the prisons. No information about the availability of Post-Exposition Prophylaxis was provided to inmates in Belgian prisons and was very infrequent in Austria. Instead such information was available in most Italian prisons. ARV were accessible in all prisons in all countries, except Italy, where only 89 % of prisons provided ARV. Interventions for the prevention of transmission through tattooing or piercing was never or rarely implemented in prisons in the four countries studied.

**Fig. 1:**
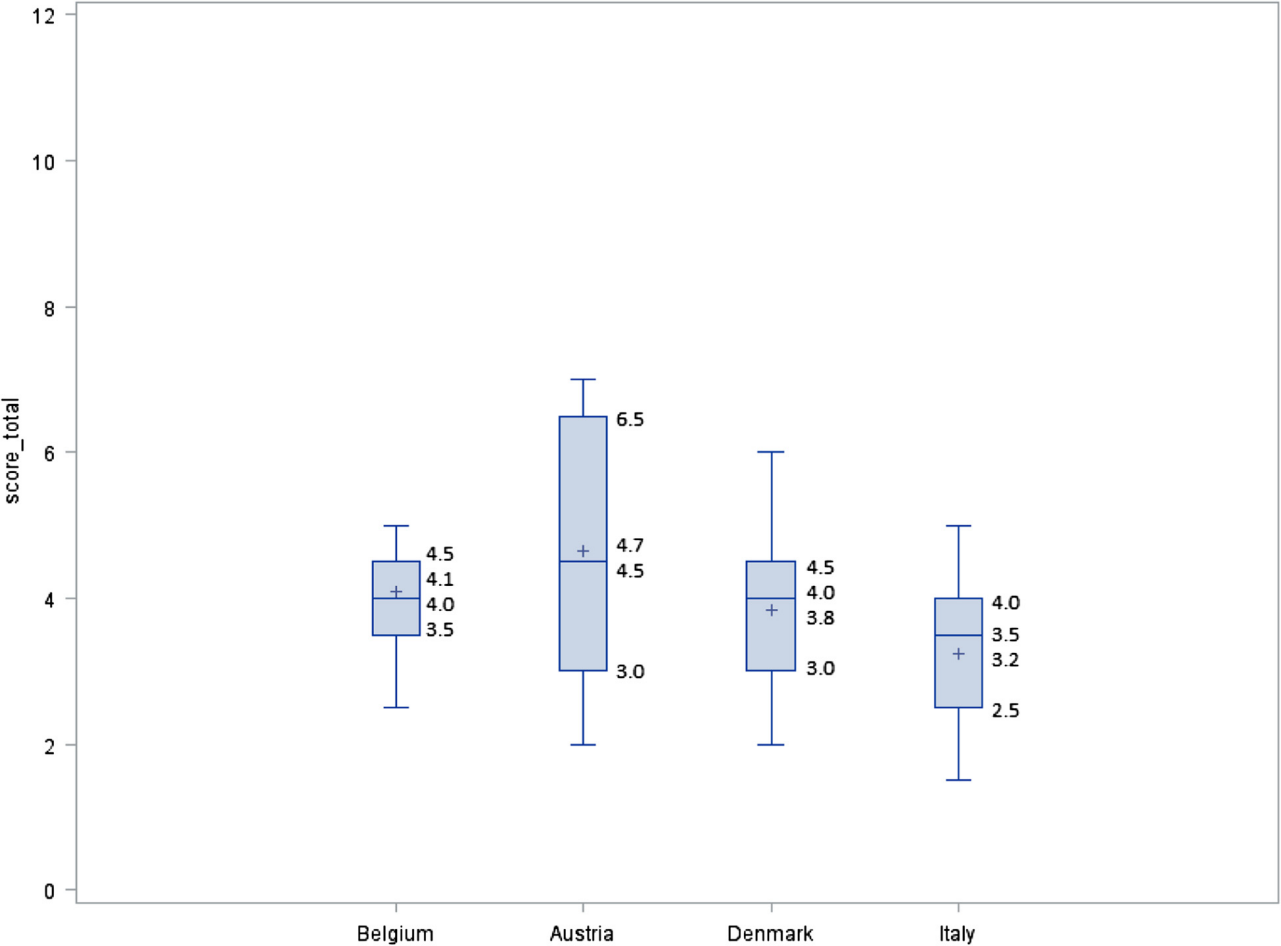
Mean, median, min and max values and interquartile range of the global score per country (E4 analysis)

At the prison level, in the E4 analysis, the adherence scores to international recommendations ranged from 0.5 to 7.0, with a maximum theoretical score of 12. Figure 1 represents the mean, median, minimum and maximum scores and interquartile range (IQR) of the global score of adherence to international recommendation per country (E4 analysis).

#### E5 analysis

**Fig. 2:**
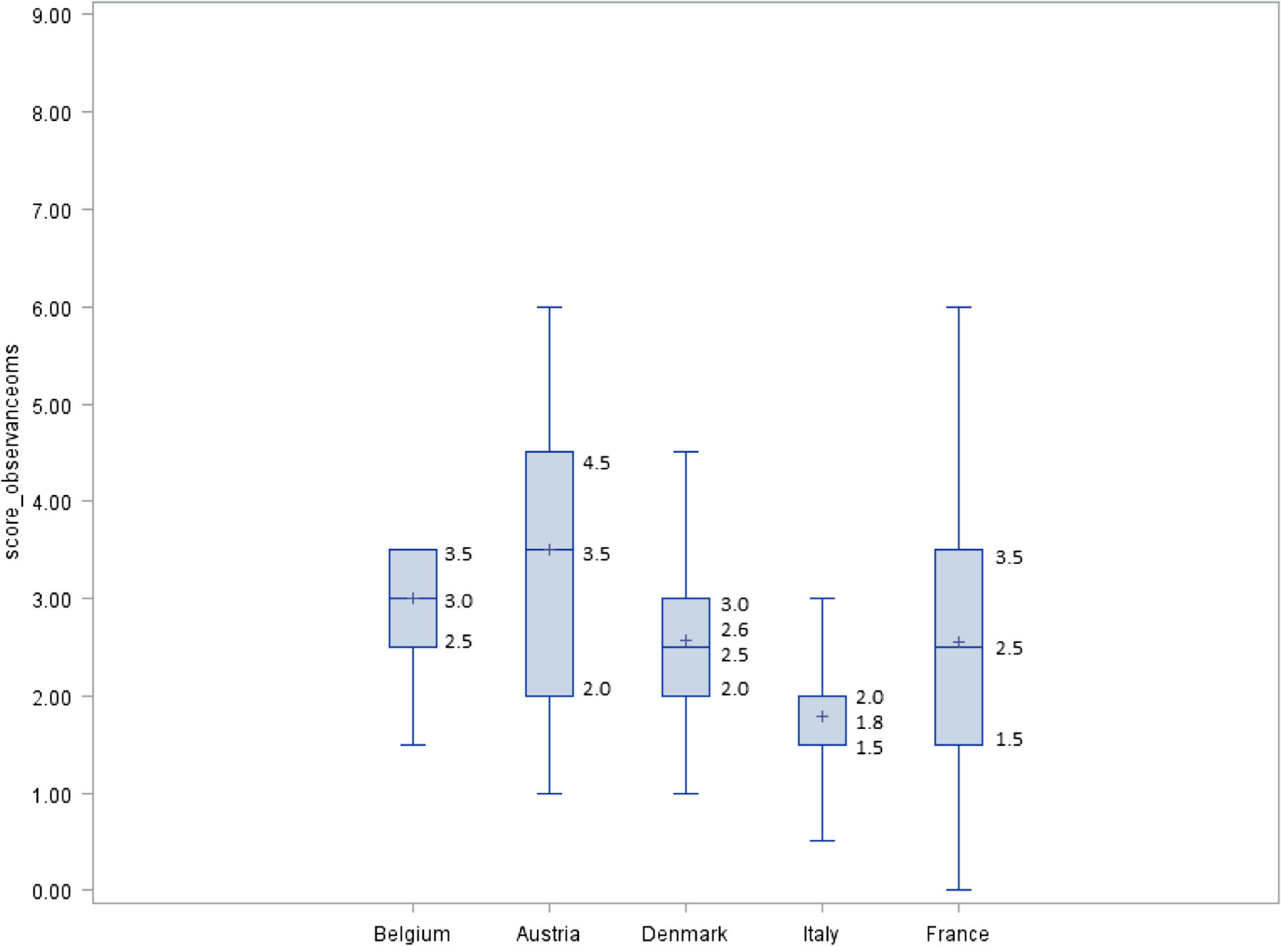
Mean, median, min and max values and interquartile range of the global score used in the 2009 French survey per country (E5 analysis)

In the 2009 French ANRS-PRI^2^DE survey, the French median [IQR] adherence score to international recommendations was 2.5 [1.5; 3.5]. After adjustment for the scoring system used in the French survey (E5 analysis), median [IQR] scores for Austria, Denmark, Belgium and Italy were 3.5 [2.0; 4.5], 2.5 [2.0; 3.0], 3.0 [2.5; 3.5] and 1.5 [1.5; 2.0], respectively (Fig. 2). Furthermore, in the E5 analysis, Austria has the highest median score (3.5, [IQR] = [3.5-6]). Denmark, France and Italy presented a significantly lower score of adherence to international recommendations when compared with Austria (Table 4).

**Table 4:** E5 analysis- univariate linear regression between country and the level of adherence to international recommendations (n = 197)

	Coef (IC95 %)	*p*
Country		
Austria	1	
Belgium	−0.50 (−1.25 ; 0.26)	*0.20*
Denmark	−0.93 (−1.52 ; −0.34)	***0.002***
Italy	−1.71 (−2.28 ; −1.14)	***<10*** ^***−3***^
France	−0.95 (−1.45; −0.45)	***0.0002***

Bold numbers have statistical significance

#### Regressions analysis

With respect to prison characteristics (Table 5), the univariate linear regression in the E4 analysis shows that in Belgium, the lower the number of security staff per prisoner, the higher the score of adherence to the international recommendations.

**Table 5:** E4 analysis- univariate linear regression between prison characteristic and the level of adherence to international recommendations

	Belgium (N = 11)		Austria (N = 19)		Denmark (N = 29)		Italy (N = 35)	
	Coef (IC95 %)	*P*	Coef (IC95 %)	*P*	Coef (IC95 %)	*P*	Coef (IC95 %)	*P*
Number of prisoners on the day of the study/100	0.03 (−0.14;0.19)	*0.72*	0.14 (−0.15;0.44)	*0.33*	0.06 (−0.31;0.45)	*0.73*	−0.01 (−0.09;0.08)	*0.85*
Number of personnel available/number of prisoners on the day of the study								
Sanitary staff	0.09 (−27.20;27.38)	*0.99*	5.94 (−2.03;13.91)	*0.13*	−0.14 (−13.33;13.05)	*0.98*	2.36 (−3.17;7.89)	*0.39*
Doctors (*100)	−0.71 (−1.48; 0.06)	*0.07*	0.43 (−0.05;0.91)	*0.08*	−0.01 (−0.24;0.22)	*0.89*	0.03 (−0.04;0.12)	*0.42*
Full time doctors (*100)	--		--		0.40 (−1.12;1.93)	*0.59*	--	
Nurses (*100)	−0.05 (−0.43;0.33)	*0.75*	0.10 (−0.02;0.22)	*0.11*	0.02 (−0.28;0.33)	*0.89*	0.05 (−0.09;0.20)	*0.47*
Full time nurses (*100)	--		--		0.09 (−0.33;0.51)	*0.66*	--	
Security staff	**−5.67 (−10.92;-0.43)**	***0.04***	6.47 (−0.75;13.70)	*0.07*	−0.71 (−2.09;0.66)	*0.30*	−0.18 (−1.15;1.52)	*0.78*
Social workers	−10.25 (−37.30;16.80)	*0.41*	52.63 (−16.04;121.30)	*0.12*	−5.31 (−25.96;15.32)	*0.60*	−5.37 (−54.13;-43.57)	*0.82*
Available Consultation								
HIV (yes vs. no)	−0.11 (−1.47;1.26)	*0.86*	--		−0.67 (−2.86;1.51)	*0.53*	0.17 (−0.71;1.05)	*0.70*
HCV/HBV (yes vs. no)	−0.11 (−1.47;1.26)	*0.86*	--		−0.67 (−2.86;1.51)	*0.53*	−0.30 (−1.17;0.57)	*0.49*
Psychiatric (yes vs. no)	−1.00 (−2.67;0.68)	*0.21*	0.18 (−2.38;2.74)	*0.89*	−0.32 (−1.48;0.83)	*0.60*	--	
Attendance of any NGO (yes vs. no)	−0.41 (−1.48;0.65)	*0.39*	0.70 (−1.24;2.63)	*0.46*	--		−0.55 (−1.18;-0.08)	*0.08*
Number of different prison types								
1								
2	−0.14 (−1.65;1.37)	*0.83*	−0.87 (−2.86;1.11)	*0.36*	−0.15 (−1.07;0.77)	*0.74*	--	
3	−0.14 (−1.65;1.37)	*0.83*	−1.56 (−3.20;0.08)	*0.06*	−0.40 (−2.65;1.85)	0.72	--	
Mixed male/female prison								
No	1							
Yes	0.02 (−0.67;0.71)	*0.95*	**−1.72 (−2.52 ;-0.92)**	***<10*** ^***−3***^	0.08 (−0.62;0.78)	*0.81*	−0.50 (−1.13;0.13)	*0.11*
Type of prison								
RC	1			*1*	1		1	
PS	−0.13 (−0.87;0.62)	*0.73*	0.70 (−0.40;1.81)	*0.21*	0.23 (−0.60;1.06)	0.58	0.07 (−0.82;0.95)	*0.88*
SPS	−0.13 (−1.27;1.02)	*0.82*	--	*--*	−0.05 (−0.93;0.82)	0.90	--	*--*
JUV	--	--	0.27 (−1.05;1.58)	*0.68*	--	--	--	*--*

HIV: Human Immunodeficiency Virus; HCV: Hepatitis C Virus; HBV: Hepatitis B Virus; NGO: Non-Governmental Organization; RC: remand center; PS: prison for sentenced; SPS: Security prison for sentenced; JUV: juvenile prison center

Bold numbers have statistical significance

In Austria, mixed prison institutes with separate male and female areas had a higher level of adherence (coefficient (95 % CI) = −1.72 (−2.52 ; −0.92); p < 10-3) than their single gender counterparts. The type of institute (remand centre, prison, security prison, juvenile prison centre) was not associated with adherence level in any country.

Considering data extracted from the Council of Europe’s annual penal statistics, one national characteristic which was moderately associated (p = 0.08) with adherence to international recommendations (5 countries analysis) was penitential density per 100 places (Table 6). The higher the penitential density per 100 places (coefficient (95 % CI) = −0.02 (−0.04; −0.002); p = 0.08), the lower the score of adherence to international recommendations.

**Table 6:** Univariate linear regression between national penal characteristics (Council of Europe’s annual penal statistics) and the level of adherence to international recommendations (5 countries analysis)

	Coef (IC95 %)	*p*
Penitential density for 100 places	−0.02 (−0.04 ; 0.002)	*0.08*
Percentage of prisoners primarily sentenced for drug offences	−0.02 (−0.08 ; 0.03)	*0.33*
Number of prisoners per security staff member	0.44 (−0.51 ; 1.39)	*0.37*
Number of prisoners per other professional	−0.01 (−0.04 ; 0.01)	*0.22*

## Discussion

To our knowledge this is the first study providing an assessment of access to HIV and HCV preventive intervention measures in prison settings of different European countries. If we consider the percentages of the overall prison population covered, participation rates for this postal/electronic survey were high in all countries (100 % in Austria, 58 % in Denmark, 50 % in Belgium, 66 % in France) except Italy (17 %). The main result is that median scores of adherence to the international recommendations for HIV and HCV prevention in prison settings were low in the 4 countries assessed using data from 2013–2014, ranging from 3.5 to 4.5 out of a maximum of 12 (full adherence). Results in these countries mirror French data from 2009–2010 [6]. Certainly, infectious risk prevention does not only depend on implementing WHO/UNODC recommended interventions, but the wide gap between these recommendations and the reality of current international and local/national practice suggests that the prison setting constitutes an environment where infectious risk continues to remain high. When adjusting the scoring mechanism to match that used in the 2009 French ANRS-PRI2DE survey (E5 analysis), Austria and Belgium have a higher adherence score than France. Denmark and France score similarly. Italy has the lowest adherence score. HR interventions are not very accessible (especially HBV vaccination proposal, bleach, information about PEP availability (except in Italy), and measures to prevent transmission through tattooing or piercing). In Italy, bleach and condoms are not available anywhere. NEP are not authorized in any prison in the five countries. Associations between structural “prison” characteristics and level of adherence to international recommendations are few: in our study, only the number of security staff per prisoner was inversely associated with the adherence score in Belgium. It is difficult to conclude anything from this result except that, globally, it is impossible to identify a characteristic associated with HR policy implementation in prisons. More interestingly, the one national structural factor inversely associated with the adherence score is prison overpopulation. This result is even more worrying when we consider that prison overpopulation is also associated with a high suicide rate [12] and increased incidence of certain diseases (e.g. tuberculosis) [13]. Overpopulation has already been associated with reduced access to care [14–17]. One reason for this is probably related to the fact that security concerns prevail over health in overpopulated prisons. Moreover, overpopulation also reflects the orientation of the global penal policy of a specific country, perhaps more focused on repression than on access to care and prevention for offenders, in particular those who use drugs. Different studies have already shown that repressive policies expose drug users to HIV/HCV infection [18–24].

Considering each prevention measure individually, it is obvious that irrespective of the country, there is an urgent need to increase the availability of most interventions. Some existing interventions differ from those implemented in the general community, and seem specific to the prison setting. One example is OST (prescribing ceiling dosages, buprenorphine crushing/dilution mainly to avoid misuse by inmates). Though not at all available in Italy, bleach is provided in all Belgian prisons, but only at one location. Intelligible information for inmates regarding the use of bleach for HR purposes is very limited everywhere. It is important to note that in the 2012 UNODC recommendations [11], providing bleach was not one of the 15 “key interventions” to prevent HIV. If bleach is to be used to efficiently disinfect syringes and needles in order to prevent HIV infection in prisons, a standardized, recognized protocol is needed. Yet the steps required to bring about the implementation of such a protocol are difficult, given the current reality of prison life and promiscuity, and would take a long time to put in place. Furthermore, bleach is not effective in preventing HCV transmission [25]. In the 2007 WHO/UNODC document, bleach was only considered as an alternative to NEP when the latter was not available. Another concern is that even though our results showed that coverage for antiretrovirals was acceptable, some prisons still have only limited access (ARV are not accessible in 10.8 % of Italian prisons,) or provide prescriptions which do not follow the corresponding national guidelines (17.6 % of prisons in Belgium, 5.7 % in Italy and 3.6 % in Austria). Tattooing and piercing are common practices in prison settings all over the world and have been clearly associated with HCV transmission [26–29]. However, associated preventive measures are very rare in the 4 countries (i.e. excluding France) assessed. Previous experience highlights the need to develop such measures [30, 31].

### Limitations

Some limitations need to be acknowledged. Although self-reports and interviews serve as the only current feasible methods to study access to prevention, they may be affected by social desirability bias. However, while the risk of over-reporting availability of preventive measures was high in our study, adherence rates were low, particularly in Italy, the country with the lowest participation rate. True scores may be even lower if we consider that the prisons included in the study may perhaps be those most active in prevention/harm reduction issues, reflected in health staffs’ decisions to answer the study questionnaire. In contrast, global coverage of the prison population and geographical representativeness were satisfactory in the survey. Second, it is also possible that physicians may not have been sufficiently informed about the interventions promoted by the prison administration in which they worked, especially in countries where health is not the responsibility of the Ministry of Justice, such as Italy. This is the case for France where access to prevention measures is the responsibility of the penitentiary administration. Response rates were different across countries despite several electronic/postal reminders (from at least 1 reminder in Austria to 3 in Italy) at the national level, and follow-up calls at the prison level for prisons. Moreover, in Belgium, a strike by sanitary staff in prisons started during the data collection period and lasted for several months. As a consequence, the survey operator had to be changed (from penitentiary administration to Ghent University). In Italy, the main explanations for the low response rate were the reluctance of sanitary staff to answer the questionnaire, and the fact that the transfer of care organization from the penitentiary administration to the national health system has not yet been fully implemented. Another difficulty in Italy is linked to questionnaire data collection. In Denmark, the response rate was lower in “remand centers”. One possible explanation for this was that sanitary staff in these usually very small prisons are external consultants with very limited interventions, in contrast to detention centers where health interventions are more structured and come under the responsibility of the Ministry of Justice. Nevertheless, considering the prison environment and the design of the survey, response rates were quite high (except for Italy).

Another possible study limitation is the weight given to NEP in our scoring: when not available, IEC and testing were scored zero, as per the 2007 WHO/UNODC recommendations. Considering the low or even zero impact of bleach on HIV/HCV prevention [25] and the clear conclusions of several other studies on HCV prevention in people who use drugs, the role of OST and NEP in reducing HCV transmission in prison settings seems to be essential. Although OST alone (methadone in particular) is effective in preventing HCV [32, 33], combining OST and NEP interventions increases this effectiveness [34–37]. In a particularly high-risk environment like the prison context, NEP should be combined with OST and other interventions to prevent infectious diseases.

## Conclusions

The most important result from this study is the low level of adherence to international recommendations for prevention interventions, which in turn is a proxy of environmental risk. This risk is associated with overpopulation in prison. As long as drug use continues to be criminalized, drug use in prison (in particular drug injection) will remain a taboo subject, despite converging arguments showing the existence of associated high-risk practices. While HR interventions need to be enhanced and scaled-up in prison settings, decriminalization of drug use, or at least alternatives to prison for drug-using populations, are urgently needed to be able to guarantee prevention and care in line with international standards.

## Abbreviations

ANRS: National Agency for Research on Aids and Viral Hepatitis
ARV: Antiretroviral Treatment
BBV: Blood Born Viruses
HBV: Hepatitis B Virus
HCV: Hepatitis C Virus
HIV: Human Immunodeficiency Virus
HR: Harm Reduction
IEC: Information – Education - Communication
ILO: International Labour Organization
NEP: Needle Exchange Program
NGO: Non-Governmental Organization
OST: Opioid Substitution Treatment
PEP: Post Exposition Prophylaxis
UNAIDS: Joint United Nations Programme on HIV and AIDS
UNDP: United Nations Development Programme
UNODC: United Nations Office on Drugs and Crime
WHO: World Health Organization
